# Personality Traits, Cognitive Styles, Coping Strategies, and Psychological Impact of the COVID-19 Pandemic Lockdown on Healthy Youngsters

**DOI:** 10.3390/bs12010005

**Published:** 2021-12-24

**Authors:** Javier Rodríguez Árbol, Alberto Ruiz-Osta, Casandra Isabel Montoro Aguilar

**Affiliations:** 1 Department of Psychology, University of Jaén, 23071 Jaén, Spain; jrarbol@ujaen.es; 2 Business Department, University of Lleida, 25003 Lleida, Spain; aro10@alumnes.udl.cat

**Keywords:** neuroticism, anxiety, depression, intolerance of uncertainty, social support seeking, negative autofocus

## Abstract

The objective of the present study was to explore possible changes in the psychological wellbeing of young healthy students during the initial 14 days of the COVID-19 general lockdown that occurred in March of 2020, and if there was any relation with specific personality traits (neuroticism, psychoticism, and extraversion), cognitive styles (internal and external locus of control and intolerance of uncertainty), and coping strategies. One hundred twenty-two university students aged from 18 to 29 years participated in the study. The dispositional factors were assessed at the beginning of the study, while measures of psychological adjustment (anxiety, depression, and self-perceived health) were taken in three different assessment stages, employing validated questionnaires and scales. Anxiety and depression scores significantly increased after one week of lockdown, reaching a plateau pattern by the second week. The levels of self-perceived mental health, vitality, and quality of life showed a pattern of sustained progressive decrease, with a more acute lessening during the first week. Neuroticism, intolerance of uncertainty, and negative autofocus were associated to worse levels of psychological adjustment. These individual differences might be taken into consideration when designing prevention programs aiming to dampen the psychological impact of a general lockdown in healthy population.

## 1. Introduction

COVID-19 has been considered to have a higher incidence in elderly people, and the increased risk of mortality in hospital has been unquestionably associated with advanced age [1,2]. Nevertheless, youngsters have also been struck by the effects of the pandemic and the associated social restrictions, especially regarding daily life activities and routines. This unexpected changes might have an impact on the wellbeing and mental health levels of otherwise healthy subjects [3,4,5].

In Spain, the restraints initially consisted of a two-week general lockdown proclaimed under a state of emergency and ordained on 14 March 2020, with the explicit prohibition of abandoning the residence for any other reason than acquiring basic supplies (food and medicaments). With the aim of protecting the health of the population against a fast spread of the COVID-19 and to stop the sharp rise in the number of deaths, the lockdown lasted almost three months and further included the closure of schools, universities, and non-essential industries, offices, and shops [6].

Subjecting the population to such a heavy social constraint could have had undesired consequences for the mental health of the individuals [7]. For instance, [8,9,10] revealed an important incidence of anxiety and depression in the general Chinese population as a result of the COVID-19 pandemic, suggesting that “precise classifying of the mental health needs across populations will facilitate development of targeted psychological interventions for individuals in epidemics of emerging infectious diseases” [10]. In this context, other authors claim that the impact in the psychological wellness of a phenomenon like the COVID-19 pandemic may be moderated by personality traits and cognitive styles, pointing to emotional lability and the proneness to experience negative affect as the “strongest predictors of clinically relevant emotional problems” [11].

This suggestion is also supported by previous research that identified the neuroticism personality trait as an important moderator of the socio-economic effects of a global crisis on the psychological wellbeing of the general population [12]. Previous research has also pointed out the role of neuroticism-related personality factors for some individuals developing anxiety in social restricted environments: professional divers who manifested anxiety levels of clinical relevance during long-term confinement in hyperbaric chambers scored significantly higher in low self-control and emotional instability than those who did not [13]. Some of the features of this kind of confinement in hyperbaric chambers, which may last for several weeks, are similar to those of the COVID-19 general lockdown. However, the latest did not depend on the willingness of the population and was accompanied by a global healthcare crisis, an unusual increase of the mortality rate, an interruption of the ordinary social life, a modification of the occupational routines, and massive damage to the world economic network. Therefore, the context of the study was also characterized by several features of traumatic nature, including sudden and unforeseen changes that could significantly increase the levels of stress of the general population, together with high levels of generalized uncertainty and a widespread lack of sense of control about the development of the situation.

The influence of personality traits and coping strategies as moderators of the psychological impact caused by traumatic and incontrollable stressful situations was also previously explored before the COVID-19 outbreak. For instance, reduced levels of optimism and reduced proneness to employ pro-active coping strategies were related to higher levels of psychological maladjustment in the face of the 1999 Kosovo crisis [14], when the psychological impact of the stressful and traumatic experiences was measured through the Brief Symptom Inventory (BSI; [15]). In the context of COVID-19 pandemic, [16] found a significant association between lower emotional stability and worse emotional response to the lockdown in the Italian general population.

Another characteristic of the COVID-19 pandemic and its collateral socioeconomic effects was the difficulty to foresee the consequences and development of the multilevel crisis, which might have supposed a high level of exposure to uncertainty for most of the general population. Therefore, specific cognitive styles, such as intolerance of uncertainty [17] or external locus of control [18], might also be involved in the relation between all the undesirable and unforeseen changes that were taking place in the social environment and the mental health of the population. Intolerance of uncertainty (IU) has been defined as a “cognitive bias that affects how a person perceives, interprets, and responds to uncertain situations on a cognitive, emotional, and behavioral level” [19]. This bias facilitates the evaluation of uncertain situations as stressful and disturbing, the avoidance of unexpected events for considering them undesirable and unpleasant, as well as the belief that not having certainty about the future is unfair [19]. Several studies [20,21] have provided evidence that supports the hypothesis of IU as being a vulnerability factor for developing anxiety disorders and depression, influencing the appraisal of ambiguous information as more threatening than it actually is and intensifying the undesirable psychological impact of coping with ambiguity and unpredictable events. In turn, locus of control (LOC) refers to “subjective appraisal of factors that account for the occurrence of events and outcomes” [22] and is usually conceptualized as a dimensional construct with two poles (Internal-External). According to the literature, people with a predominant internal orientation consider the outcomes of events to be contingent upon their own actions, whereas individuals with a predominant external orientation event view outcomes as largely influenced by outside forces, such as other people and chance [18,23]. Concerning LOC as an individual dispositional factor, high scores on external LOC have been associated with symptoms of depression and anxiety in western cultures [22].

As was shown in [14], besides the interpretative framework that provides the previous research on personality traits and cognitive styles, the possible influence that certain behavioral patterns (especially if they are recurrent) might have as protective factors for the development of anxiety, depression, and other health problems when dealing with stressful situations and crisis is also of interest [24]. The concept of coping strategies refers to “cognitive and behavioral efforts to master, reduce, or tolerate” the demands emerging in these challenging contexts [25], and are characterized by their function (i.e., seeking social support, emotional discharge, problem solving, self-isolation, emphasizing the positive…), instead of their outcome, and therefore can be clustered according to the former criterion. The empirical research and subsequent factorial analysis allowed to group the different strategies in two higher-order coping dimensions: problem-focused and emotional-focused coping [26]. Although there is a general consensus about the adequacy of considering the two former factors [24], some authors have proposed to add any extra major dimension, e.g., avoidance [27] and religion [28]. Regarding the role of coping strategies as moderators of the impact caused by stressful situations, previous research found an association between individual predominance of avoidance and emotional-focused type strategies and poorer health and increased negative affect among college students [29,30,31], whereas problem focused coping styles like cognitive reinterpretation and problem solving strategies were associated to better health and higher wellbeing levels [32]. 

In summary, besides the medical conditions and mortality directly caused by the SARS-CoV-2 virus, the COVID-19 pandemic generated global healthcare, economic, and social crises that might have caused an important impact on the mental health of the population, even for those people not directly affected by the virus. Additionally, this impact might be moderated by individual features such as those previously presented. 

### Objectives of the Study

Given this exceptional situation, the present study aimed to address two main objectives: 1. Assessing the possible development of anxiety and depression related symptoms during the initial two-weeks general lockdown, as well as possible fluctuations in well-being and self-perceived health, in a sample of young healthy people with no previous mental conditions. 2. Identifying whether certain psychological dispositional factors (level of neuroticism as personality trait, IU and LOC as cognitive styles, and the predominant coping strategies) would be associated with the dynamics of the former variables. According to the literature [13,21,22,29], those people who scored higher in neuroticism and IU showed an external LOC and predominantly reported avoidance and emotion-focused coping strategies (i.e., negative autofocus or overt emotional expression) would be more prone to experience higher levels of anxiety and depression symptoms during the general lockdown, as well as to report lower levels of self-perceived health.

## 2. Materials and Methods

### 2.1. Participants

A total of 210 healthy young Spaniards, all of them psychology undergraduates, were asked to fill in a set of questionnaires and to complete a weekly tracking assessment during the two first weeks of lockdown. The contact was made through the virtual learning environment of the University of Jaen. Up to 165 of them voluntarily accepted to participate in the study and to facilitate their data to the researchers. The agreement to participation was explicitly established by signing an informed consent. Exclusion criteria comprised being under 18 or above 30 years old, the diagnosis of mental disorder, and the consumption of any of the following substances during the two weeks of assessment: anxiolytics, antidepressants, hypnotics, benzodiazepines, and any type of illegal psychoactive substances. Hence, ten participants had to be excluded from the original sample, five of them for having reported suffering from a mental condition (three of them declared having depression, one informed of a non-specified anxiety disorder, and a last one declared suffering from panic disorder), and another five for having consumed any of the substances included within the exclusion criteria. Moreover, the data of fifteen people were removed from the analysis because of the age exclusion criterion. Eventually, eighteen participants did not fully complete any of the assessment tools nor provide a valid file with their answers, so they were also removed from the dataset.

The final sample was composed by 122 university students (of which 96 were females and 26 were males) aged from 18 to 29 (M = 20.87, SD = 2.21). None of them was suffering from any mental condition nor had they consumed any illegal drug that could significantly affect their emotional state during the lockdown.

#### Power Analysis and Sample Estimation

The power analysis made by means of Gpower3 [33] estimated a sample size of 89 participants for 1 − β = 0.95, α = 0.05 and an expected medium effect size [34].

### 2.2. Psychological Measures

All the measures were taken employing standardized and validated questionnaires. For the assessment of the predominant personality traits, every participant completed the Spanish Adaptation of the Eysenck Personality Questionnaire-Revised: EPQ-R. The EPQ-R was developed by Eysenck, Eysenck, and Barrett [35] and translated into Spanish by Eysenck and Eysenck [36]. It consists of 83 items spread among four subscales, namely neuroticism, extraversion, psychoticism, and social desirability (Sincerity). Scores range between 0 and 23 on all subscales (answer format = YES/NO). Internal consistency (Cronbach’s α) for all scales ranged from 0.81 to 0.82 [37]. 

The information about the individual cognitive styles was gathered by means of two standardized questionnaires, aimed to assess the IU and the LOC. For the first construct, the Spanish version of the Intolerance of Uncertainty Scale was employed: IUS [17]; Spanish adaptation: [38], a Likert type questionnaire with 27 items, subdivided in two main factors: inhibition eliciting uncertainty (IGI, 16 items) and uncertainty as confusion and unexpectedness (IDI, 11 items)). The latest authors reported an internal consistency (Cronbach’s α) of 0.91 (0.93 for IGI and 0.89 for IDI). 

For assessing the LOC, the Spanish version of the Locus of Control Scale (LCS) [39,40] originally introduced as I-E Scale by Rotter was employed [18]. It consists of 29 items with dichotomous response choices and provides a score that indicates the predominant locus of control (internal or external) of the individuals. Internal consistency (Kuder-Richardson test for measures with dichotomous choices) in samples of college students ranged from 0.69 to 0.76 [18]. A more recent reliability generalization study provided an overall estimate of the internal consistency score reliability of 0.71 [41]. 

The predominant strategies of management of stressful situations were assessed through the Stress Coping Questionnaire: CAE [28]. The CAE is a self-administered Likert scale of 42 items that assesses 7 basic coping styles: problem solving focus, negative autofocus, positive re-evaluation, overt emotional expression, avoidance, social support seeking, and religion. Internal consistency (Cronbach’s α) for the 7 subscales ranged from 0.64 (negative autofocus) to 0.92 (social support seeking), with a mean α score of 0.79 in Spanish population [28]. The second-order factor analysis suggested the existence of two higher-order dimensions that might group five of the seven factors: problem-focused strategies (problem solving focus, positive re-evaluation, and social support seeking) and emotion-focused strategies (overt emotional expression and negative autofocus), whereas avoidance and religion did not fit in any of the higher-order dimensions [28].

The psychological well-being and its fluctuations were assessed by the Spanish adaptation [42] of the Short-form Health Survey: SF-36 [43,44,45]. This instrument comprises 36 items that assess positive and negative health states. It provides direct scores for 8 scales that represent the most frequently employed health, illness, and treatment related concepts: Physical Functioning, Role-Physical, Bodily Pain, General Health, Vitality, Social Functioning, Role-Emotional and Mental Health. Additionally, the SF-36 allows to obtain a general score related to Global Quality of Life. A meta-analysis that examined the psychometric properties of the Spanish adaptation of the instrument [46] provided internal consistency coefficients (Cronbach’s α) above 0.70 for all the scales, with Cronbach’s α ≥ 0.90 for Physical Functioning, Role-Physical, and Role-Emotional. For all the scales, the scores are directly related with well-being, so higher scores denote a more positive self-perception of one’s health in the respective area. 

The level of anxiety was assessed with the Spanish adaptation of the State-Trait Anxiety Inventory: STAI [47]. It consists of 40 items divided in two subscales with 20 items each: State Anxiety and Trait Anxiety. The inventory is scored using a Likert scale of four response alternatives, (score range: 0–60, for both state and trait anxiety). The internal consistency of the Spanish version ranges between 0.90 and 0.93 for state anxiety and between 0.84 and 0.87 for trait anxiety [47]. A study conducted in 2011 that analyzed the properties of the test employing a sample of Spanish population (N = 1036) provided an internal consistency of 0.90 for trait anxiety and 0.94 for state anxiety [48]. 

The depression symptoms were assessed by the Spanish adaptation of the Beck’s Depression Inventory II: BDI-II [49]. The BDI-II consists of 21 items rated on 4-point Likert scales according to symptom severity (score range: 0–63). The internal consistency of the Spanish version ranges between 0.76 and 0.95. The score obtained in the inventory is directly related to the presence and severity of depressive symptoms. Previous research with the Spanish population suggests a cut score of 14 points for clinically relevant depression with mild symptomatology [50].

### 2.3. Procedure 

The first day of the lockdown (A1), the participants were asked to answer all the above informed questionnaires and to write down their direct responses in a text editor file. Instructions were given for generating a personal alphanumeric identification code for the text file, thus allowing to perform follow-up during the two weeks of the state of emergency while maintaining the anonymity of the participants.

**Figure 1:**
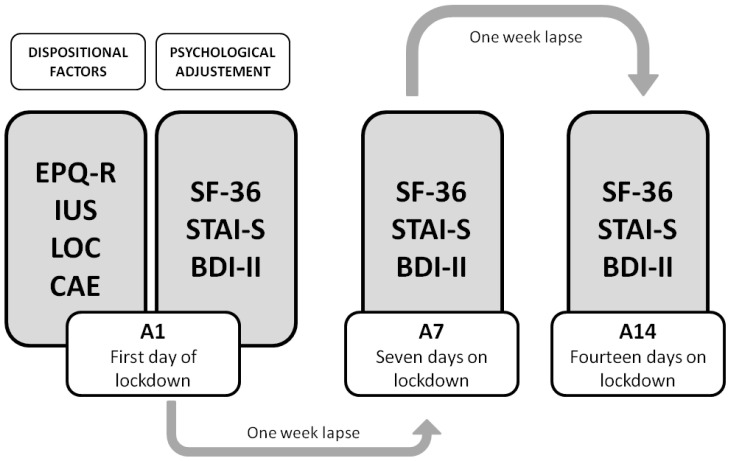
Procedure of the study, with the three assessment stages: A1, A7, and A14.

After seven days (A7) and two weeks (A14) of lockdown, the participants were asked to again answer the STAI-State (STAI-S), BDI-II, and SF36 (see Figure 1 for better comprehension of the procedure).

Once the two weeks of assessment had finished, the participants uploaded their text files with their direct answers to a closed virtual platform, using their personal alphanumeric codes to name their files.

### 2.4. Data Analysis

The direct answers to the questionnaires given by the participants were introduced into a data base by an instructed researcher, providing a digital anonymous code for each participant. Then, the direct scores were obtained for every measure and participant, by means of a series of algorithms built ad-hoc for this study, which followed the standard correction rules for the respective measures. Prior to launching the analysis, the data were visually inspected in order to detect missing inputs or errors, discarding those records that were incomplete or that presented any type of anomaly in the input format.

The data were analyzed in two stages with the following objectives: (1) Identifying any possible fluctuation in the variables related to mental-health and wellbeing (measured through BDI-II, STAI-S, and SF-36) during the two first weeks of the general lockdown; (2) Identifying possible correlations between the selected individual dispositional factors (measured through EPQ-R, IUS, LOC, and CAE) and the dynamics of the previously mentioned variables.

In the first stage, the data were analyzed by means of a multivariate analysis of variance (MANOVA) with the within-subjects factor “Time” (with three levels: A1, A7, and A14), including the scores obtained in BDI-II, STAI-S, and SF-36 scales in the three different assessment times. Sphericity assumption was tested by means of Mauchly’s sphericity test and Greenhouse–Geisser correction was applied when this assumption was violated. The results of the MANOVA are presented by reporting the original degrees of freedom and the corrected *p*-values, together with the effect size. Two different estimates of effect size are reported for the univariate tests: generalized eta squared (ηG2) to facilitate across-studies interpretations and meta-analyses [51], and partial eta squared (ηp2) to interpret the results according to Cohen’s benchmarks [34]. Significant effects were analyzed using *t*-tests for pairwise comparisons applying Bonferroni correction.

Then, correlation analyses were conducted for the mean of the direct scores in the three assessment stages of the psychological adjustment variables, which included state-anxiety (STAI-S), depression symptoms (BDI-II), and self-perceived health (SF-36, only the scales that showed significant variations along the two weeks of assessment were included in the correlation analyses: Vitality, Mental Health, Role Emotional, and Global Quality of Life), and the scores of the individual dispositional factors: personality traits, cognitive styles, and coping strategies (obtained by EPQ-R, IUS and LCS, and CAE, respectively).

In order to explore possible associations between the dynamics of psychological adjustment and the measured dispositional factors, the differential scores between the respective assessment stages (A7−A1; A14−A7; A14−A1) were also obtained for the former mentioned variables and included in the analyses.

Prior to launching the correlation analyses, the normality assumption was checked for all the measures by means of the Shapiro–Wilk normality test. Given that the normality assumption was violated in eleven out of nineteen variables, Spearman’s rho (rs) the correlation coefficient was estimated. 

MANOVA analyses were performed employing IBM SPSS15 (SPSS Inc.: Chicago, IL, USA), while the correlation analyses and the normality assumption tests were performed employing the open software JASP [52]. The dataset has been posted in a permanent public repository with open access and can be downloaded from this URL: https://osf.io/pnzjv/?view_only=9deaa3ff4953423bb434ac67f146a0ae (accessed on 25 March 2021).

## 3. Results

The MANOVA showed a significant within-subject effect of the factor “Time” (Wilk’s lambda = 0.768, F(24, 462) = 2.71, *p* < 0.001, ηp^2^ = 0.124). The result of Mauchly’s sphericity test was statistically significant for all the variables (all *p* values < 0.01), except for STAI-S (*p* = 0.094) and SF-36′s Pain scale (*p* = 0.564). The univariate analyses, which were consequently performed applying Greenhouse–Geisser correction, provided the following results:

### 3.1. Depression Symptoms (BDI-II) 

There was found to be a significant difference in depression symptoms along the two weeks (F(2, 242) = 7.784, *p* = 0.001, ηG^2^ = 0.007, ηp^2^ = 0.06). The BDI-II score increased an average of 1.7 points after one week of lockdown (A7: M = 13.72, SD = 9.48) in comparison with the scored registered on the first day of lockdown (A1: M = 12.02, SD = 9.45). The BDI-II score on the 14th day (A14: M = 13.65, SD = 8.997) was slightly lower than that on A7. The post-hoc analysis indicated that BDI-II scores increase was statistically significant from the first day of lockdown to the 7th (*p* = 0.002), meanwhile the differences between A7 and A14 were not statistically significant (*p* = 1.00). Nevertheless, the BDI-II score on A14 was still significantly higher than that at A1 (*p* = 0.012).

### 3.2. State-Anxiety (STAI-S)

The univariate analyses showed a significant difference in state-anxiety along the two weeks (F(2, 242) = 5.794, *p* = 0.004, ηG^2^ = 0.016, ηp^2^ = 0.046). As occurred with depression levels, the same pattern of evolution was observed for the STAI-S scores: the STAI-S score significantly increased (*p* = 0.013) from A1 (M = 25.47; SD = 12.8) to A7 (M = 28.94; SD = 11.95). A slight decrease of the STAI-S score was oppositely observed between the 7th and the 14th day, but this last difference was not statistically significant (*p* = 1.00). The STAI-S score on A14 (M = 28.67; SD = 12.87) was still significantly higher than the first day of lockdown (*p* = 0.024).

### 3.3. Self-Perceived Health (SF-36)

There were also found to be significant differences in three out of eight SF-36 scales: Mental Health (F(2, 242) = 14.765, *p* < 0.001, ηG^2^ = 0.019, ηp^2^ = 0.109), Vitality (F(2, 242) = 12.841, *p* < 0.001, ηG^2^ = 0.015, ηp^2^ = 0.096), and Role-Emotional (F(2, 242) = 3.847, *p* = 0.03, ηG^2^ = 0.007, ηp^2^ = 0.031); as well as in the global score of Quality of Life (F(2, 242) = 8.414, *p* = 0.001, ηG^2^ = 0.008, ηp^2^ = 0.065).

#### 3.3.1. Mental Health

Regarding the scores of the Mental Health scale, the data showed a progressive decrease in the self-perceived level of Mental Health during the lockdown, with a sharper lessening after the first week, as illustrated by the differential score of −1.07 points between A1 (M = 19.82, SD = 4.97) and A7 (M = 18.72, SD = 4.56), a difference that was statistically significant (*p* = 0.003). The score was even lower on A14 (M = 18.22, SD = 4.66), but the difference with the score registered on A7 was not significant (*p* = 0.065). Nonetheless, it still entailed a significant fall of 1.6 points below the mean of the first day (*p* < 0.001).

#### 3.3.2. Vitality

The Vitality scale followed a similar pattern of decline: a lessening from A1 (M = 14.67, SD = 3.89) to A7 (M = 13.97, SD = 3.79), reaching the lowest score at A14 (M = 13.51, SD = 3.83). Again, the decrease was more pronounced after the first week (−0.7 points) than in the second week of lockdown (−0.46 points), but in this variable both steps showed statistically significant differences (*p* = 0.008 for A1−A7, *p* = 0.035 for A7−A14).

#### 3.3.3. Role-Emotional

The Role-Emotional scale showed a decrease from A1 (M = 4.73, SD = 1.37) to A7 (M = 4.6, SD = 1.36) and kept lessening on A14 (M = 4.44, SD = 1.42). Nevertheless, the pairwise comparisons showed that only the difference between A1 and A14 was statistically significant (*p* = 0.045).

#### 3.3.4. Global Quality of Life

The global score of Quality of Life followed the same dynamics as the previous variable: a diminution from A1 (M = 115.61, SD = 14.59) to A7 (M = 113.76, SD = 13.56) reaching an even lower score on A14 (M = 112.6, SD = 13.92), the difference between A1 and A14 being the only statistically significant one (*p* = 0.001).

No significant differences were observed for the rest of the SF-36 health variables (Physical Functioning, Role-Physical, Bodily Pain, General Health, and Social Functioning).

### 3.4. Associations between Mean Scores of Depression, Anxiety, and Perceived Health and Individual Dispositional Factors 

**Figure 2:**
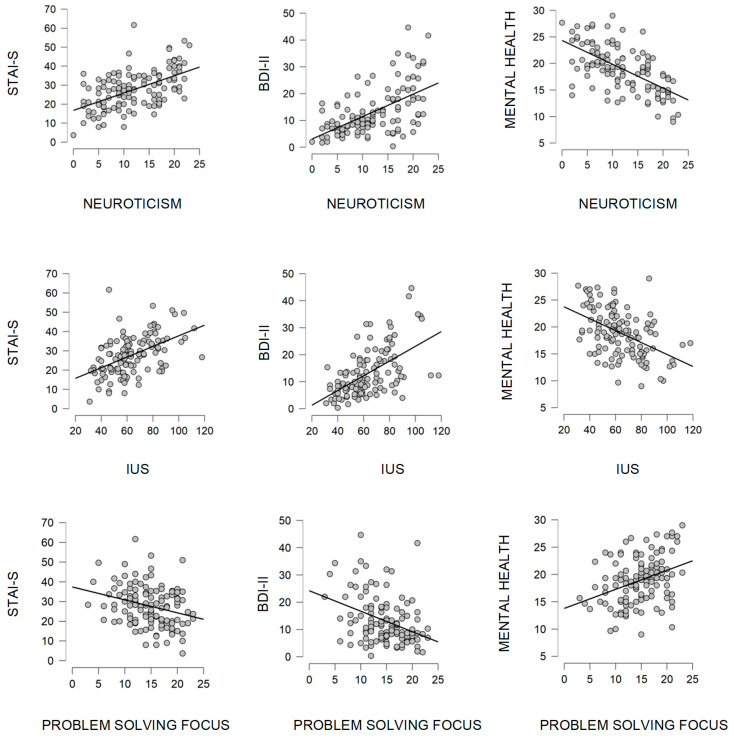
Dispersion graphs showing the two patterns of associations (IUS = Intolerance of Uncertainty).

Significant associations among the mean scores of STAI-S, BDI-II, and SF-36 scales obtained during the two-week period and the scores of the individual dispositional factors are reported in Table 1. Dispersion graphs for the most prominent associations are provided in Figure 2. There are five dispositional factors (Neuroticism (NEUR.), Extraversion (EXTR.), Intolerance of Uncertainty (IUS), Problem Solving Focus (PSF), and Negative Autofocus (NA)) that showed consistent associations with anxiety, depression, and the self-perceived health scales. For three of them (NEUR., IUS, and NA) the associations could be considered moderated (all |r_s_| > 0.4 except for those related to Role-Emotional), meanwhile for the remaining two (EXTR. and PSF) the associations should be considered significant but weak. Furthermore, there are two different patterns of association that could be clearly identified: on one hand, NEUR., IUS, and NA showed a positive association with the STAI-S and BDI-II scores, whereas inversely they showed a negative association with the SF-36 scales. On the other hand, EXTR. and PSF showed the opposite pattern: negative associations with the STAI-S and BDI-II scores along with positive associations with the SF-36 scales are observed. It is important to point out that all the dispositional factors mentioned showed a weaker association with the SF-36 Role-Emotional scale than with the rest of the variables.

The possible presence of multicollinearity was tested by means of an analysis of the correlation coefficients between the reported dispositional factors (all the |r_s_| < 0.6) and the estimation of the Variance Inflation Factor (VIF) and Tolerance (TOL) [53]. These are the obtained results: Neuroticism (VIF = 1.823, TOL = 0.549), Extraversion (VIF = 1.269, TOL = 0.788), Intolerance of Uncertainty (VIF = 1.727, TOL = 0.579), Problem Solving Focus (VIF = 1.324, TOL = 0.755), Negative Autofocus (VIF = 1.726, TOL = 0.579). According to the traditional benchmarks, these results suggest that there was no presence of strong collinearity between the reported variables.

### 3.5. Associations between Differential Scores of Depression, Anxiety, and Perceived Health in Different Assessment Stages and Individual Dispositional Factors 

There was not found to be any statistically significant correlation between the differential scores of the psychological adjustment indexes and the dispositional factors after applying Bonferroni correction for multiple comparisons.

## 4. Discussion

This study aimed to assess the level of certain psychological variables that are considered sensitive to critical changes in the social environment, as well as several dispositional factors that could moderate the psychological impact that such an experience as a global health crisis together with a general lockdown could have on young healthy individuals. Regarding the first research objective, the most salient result is the significant increase in anxiety and depression scores during the first week of lockdown (although with small-medium effect sizes [34]), together with the significant lessening of the self-perceived levels of mental health and vitality, showing the former to be the most important effect size among all the measured variables in the study. Although the interpretation of effect sizes values must be undertaken with caution [54], it is remarkable that the mean score in the BDI-II along the assessment period reached the threshold of mild-depression (established in 14 points for the local population [50]), which reflects a higher value than that observed in other studies with a Spanish sample of the same range of age [49] and those carried out with international samples of college students [55]. This fact is especially noteworthy considering that none of the participants had previous mental health issues (it was one of the exclusion criteria) and that the sample variance in BDI-II scores kept similar to those of the mentioned studies. The STAI-S mean score also ranged higher than expected in general population according to the local normative data [48]. Nevertheless, the score of both variables seemed to have reached a plateau by the second week of confinement, in the threshold of the clinical relevance, which could be a sign of a resistance to develop severe symptomatology even under extreme conditions. On the other hand, the self-perceived health indexes kept worsening in the last assessment, suggesting different dynamics. Maybe the fact that SF-36 measures are not related to any specific disorder or clinical construct (like BDI-II), but rather gather broader self-perceptions about one’s health may explain this difference in the evolution patterns. 

Besides exploring the dynamics of variables related to mental health, the current study also aimed to identify possible associations between the psychological adjustment reflected by this variables and individual dispositional factors that had been previously identified in the literature as being of influence in the face of a sudden and unforeseen change in the social environment. Nevertheless, the significant associations have been mainly found with the mean scores of the psychological adjustment variables rather than with the differential scores of the respective assessment stages. In fact, five out of twelve dispositional factors measured in the study showed a pattern of consistent associations with the mean of the psychological variables significantly affected during the lockdown, whereas none of them showed significant associations with the differential scores. The former five dispositional factors referred to personality traits (neuroticism and extraversion), cognitive styles (IUS) and predominant coping strategies (problem solving focus and negative autofocus). Moreover, two different patterns of associations can be clearly identified among the mentioned factors. The first pattern is characterized by positive correlation coefficients with both BDI-II and STAI-S scores and negative correlation coefficients with the SF-36 health scales, i.e., a configuration that could be representative of vulnerability factors, as may be appreciated in the dispersion graphs of Neuroticism and IUS (Figure 2). The second pattern, on the contrary, reflects negative correlation coefficients with BDI-II and STAI-S scores and positive associations with the SF-36 health scores. In this case, the scheme of the associations is the expected for protective factors, as can be seen in the dispersion graphs of Problem Solving Focus (Figure 2). Interestingly, although both kinds (vulnerability and protective factors) show similar consistency in their associations, the dispositional factors that display the vulnerability pattern showed higher correlation coefficients than those displaying the protective pattern. Thus, higher levels of neuroticism, intolerance of uncertainty, and negative autofocus characterized the vulnerability pattern and showed moderate levels of |rs| (with absolute values ranging from 0.4 to 0.63), while extraversion and problem solving focus showed the protective scheme of associations with overall weaker |r_s_| (with absolute values ranging from 0.25 to 0.37). 

Neuroticism showed the most consistent associations, standing out as a dispositional factor to be considered when predicting possible outcomes of social restrictions over young healthy subjects. The results are coincident with other studies conducted in other countries with different population groups [56,57,58,59] and are coherent with the framework of a transdiagnostic perspective, where neuroticism has also been one of the more often highlighted dispositional factors for the development of emotional and mood disorders, pointing out the resulting emotion dysregulation as “underlying all anxiety, depressive, and related disorders” [60]. This has been explained by the temperamental propensity to experience negative affect, the pervasive negative appraisal of their emotional experiences and context, and the persevering (and frequently unsuccessful) effort to control them [61], which characterizes the mentioned personality trait and is coherent with the associations observed in the current study. Regarding this, it is also remarkable that Negative Autofocus is the coping strategy that showed the strongest vulnerability pattern among all of the CAE factors, result that supports this perspective.

Contrary to what was expected, no associations were found among the mean scores of the psychological adjustment variables and social support seeking, a coping strategy that has been traditionally considered as emotion-oriented [24,26]. According to this classification, some studies have provided data that portray it as a dispositional factor associated to poorer health levels [32]. Nevertheless, some authors claim that the consistent tendency to seek social support should be rather conceptualized as a resource for coping strategies instead of a proper coping strategy itself [27], since it can become a facilitator in task-oriented strategies (i.e., asking for useful information that can help to solve the problem) or in emotional regulation (i.e., gathering with a close friend and talk about the issue and the feelings related), which could explain those data which suggest that social support seeking as a salient coping strategy might be a protective dispositional factor in extreme situations [14]. There is a major point concerning the current study, however, that may have had an important influence in the results: the COVID-19 general lockdown implied a severe restriction of mobility, which made it impossible to display many of the social behaviors that are usually intended to provide this kind of support and could have ultimately affected the effectiveness of these kinds of coping strategies, as was also observed in a study conducted in Italy during lockdown [62]. 

Also contrary to the initial hypotheses is the absence of significant associations with psychological adjustment for the predominance of avoidance type coping strategies, which have been portrayed in previous studies as a vulnerability factor for higher levels of stress among college students [29]. However, regarding the context of a generalized crisis, in a previous study conducted in extreme conditions (1999 Kosovo crisis) no correlations were found either between the avoidance coping style and psychological adjustment [14]. 

The correlation coefficients of external LOC are less consistent and weaker than those observed of other dispositional factors, in contrast to what has been observed in studies conducted during the COVID-19 pandemic with different populations [63,64]. For example, comparing the r_s_ values of LOC and IUS with BDI-II scores (0.22 and 0.56, respectively), it is remarkable that the latest factor showed a much stronger association with depression, which has been closely associated to external LOC [22]. These results speaks for the line of research that considers intolerance of uncertainty as an important transdiagnostic vulnerability factor, ubiquitous in anxiety and depression disorders [21].

Although contextual factors not assessed in this study, e.g., the characteristics of the dwelling where the people were confined or the precise impact of the lockdown on the family’s financial conditions, should not be disregarded, the individual dispositional factors discussed here may take account for part of the differences in the psychological adjustment experienced as a consequence of the lockdown. This may be of help in foreseeing which people are more prone to suffer an impairment of mental health due to the social and environmental restrictions that suppose a general lockdown, as well as for designing and deploying specific healthcare and psychosocial strategies aiming to dampen the psychological impact of a pandemic or similar global crises in the general population.

## 5. Limitations

The fact that the first measure was taken on the first day of the state of emergency does not allow to make a comparison with an actual base-line of the variables of interest (in the sense of having been taken under no-restriction conditions). This means that the participants were already aware of the implemented restrictions and that they were due to a national healthcare emergency situation. Therefore, it is plausible that any possible first shock effect was not gathered by our measures. The homogeneity of the sample (all undergraduate students from the same country) also implies that any possible extrapolation of these results to any other population group should be undertaken with caution.

## 6. Conclusions

A sample of healthy college students with no previous mental condition experienced a significant increase of anxiety and depression symptoms throughout the general lockdown during the COVID-19 pandemic, reaching higher levels than the average scores obtained in normative studies. However, this increase was not linear, as more acute worsening was observed during the first week, and the data showed a plateau in the psychological adjustment variables after two weeks of lockdown. The levels of self-perceived mental health, vitality, and quality of life maintained a pattern of progressive decrease, but with a more acute lessening during the first week.

The psychological dispositional factors considered as ubiquitous in the spectrum of stress and mood disorders (neuroticism and intolerance of uncertainty) correlated as expected with the dynamics of the psychological adjustment during the lockdown, showing an association pattern that points them out as possible vulnerability factors for the development of anxiety and mood disorders. The results suggest that the propensity to employ Negative Autofocus as a coping strategy could also be a vulnerability factor. Inversely, Problem Solving Focus was associated with better psychological adjustment. 

The predilection for Social Support Seeking as a usual coping strategy was slightly associated with a relative increase of depression and anxiety levels, as well as with the impairment of the self-perceived mental health as the lockdown progressed. 

These individual differences might be taken into consideration when designing prevention programs aiming to dampen the psychological impact of a general lockdown in a healthy population.

## Figures and Tables

**Table 1:** Spearman correlation coefficients (r_s_) for the mean of the psychological adjustment variables that significantly worsened during the lockdown and the individual dispositional factors.

PSYCH. ADJ.	NEUR.	EXTR.	IUS	PSF	NA
State-Anxiety (STAI-S)	0.524e *	−0.253	0.502 *	−0.279	0.551 *
Depression (BDI-II)	0.559 *	−0.353	0.558 *	−0.368 *	0.634 *
Vitality (SF-36)	−0.521 *	0.339 *	−0.429 *	0.364 *	−0.550 *
Mental Health (SF-36)	−0.617 *	0.277e	−0.450 *	0.358 *	−0.579 *
Global Quality of life (SF-36)	−0.462 *	0.278	−0.419 *	0.373 *	−0.479 *

Note: * *p* < 0.05 after applying Bonferroni correction for multiple comparations. EPQ-R personality traits: neuroticism (NEUR.), extraversion (EXTR.). Cognitive styles: Intolerance of Uncertainty (IUSe). Coping strategies: problem solving focus (PSF) and negative autofocus (NA). PSYCH. ADJ. = Psychological Adjustment variables.

## Data Availability

The authors claim that this manuscript describes an original research work which has not been preregistered. The dataset related to this research is available at a permanent public repository with open access. https://osf.io/pnzjv/?view_only=9deaa3ff4953423bb434ac67f146a0ae (accessed on 25 March 2021).
